# Miniaturization optimized weapon killing power during the social stress of late pre-contact North America (AD 600-1600)

**DOI:** 10.1371/journal.pone.0230348

**Published:** 2020-03-17

**Authors:** Anna Mika, Kat Flood, James D. Norris, Michael Wilson, Alastair Key, Briggs Buchanan, Brian Redmond, Justin Pargeter, Michelle R. Bebber, Metin I. Eren

**Affiliations:** 1 Department of Anthropology, Kent State University, Kent, Ohio, United States of America; 2 School of Anthropology and Conservation, University of Kent, Kent, United Kingdom; 3 Department of Anthropology, University of Tulsa, Tulsa, Oklahoma, United States of America; 4 Department of Archaeology, Cleveland Museum of Natural History, Cleveland, Ohio, United States of America; 5 Department of Anthropology, New York University, New York, New York, United States of America; 6 Archaeology and Environmental Studies, School of Geography, Rock Art Research Institute, University of the Witwatersrand, Johannesburg, South Africa; Ghent University, BELGIUM

## Abstract

Before Europeans arrived to Eastern North America, prehistoric, indigenous peoples experienced a number of changes that culminated in the development of sedentary, maize agricultural lifeways of varying complexity. Inherent to these lifeways were several triggers of social stress including population nucleation and increase, intergroup conflict (warfare), and increased territoriality. Here, we examine whether this period of social stress co-varied with deadlier weaponry, specifically, the design of the most commonly found prehistoric archery component in late pre-contact North America: triangular stone arrow tips (TSAT). The examination of modern metal or carbon projectiles, arrows, and arrowheads has demonstrated that smaller arrow tips penetrate deeper into a target than do larger ones. We first experimentally confirm that this relationship applies to arrow tips made from stone hafted onto shafts made from wood. We then statistically assess a large sample (n = 742) of late pre-contact TSAT and show that these specimens are extraordinarily small. Thus, by miniaturizing their arrow tips, prehistoric people in Eastern North America optimized their projectile weaponry for maximum penetration and killing power in warfare and hunting. Finally, we verify that these functional advantages were selected across environmental and cultural boundaries. Thus, while we cannot and should not rule out stochastic, production economizing, or non-adaptive cultural processes as an explanation for TSAT, overall our results are consistent with the hypothesis that broad, socially stressful demographic changes in late pre-contact Eastern North America resulted in the miniaturization–and augmented lethality–of stone tools across the region.

## Introduction

The archaeological record for the late first millennium AD reveals that native societies across the North American midcontinent and northeast initiated a suite of cultural changes that culminated in the development of sedentary, maize agricultural lifeways of varying complexity. Among the most important elements of this culture change were increased population nucleation, intergroup conflict (warfare), the adoption of the bow and arrow, and increased territoriality [1,2]. This process climaxed with the development of Mississippian chiefdoms as early as AD 1050 in the American Bottom [3] and tribal-level peer-polities after about AD 1300 [4–6]. Increased population and territoriality is revealed in changing site distributions marked by the clustering of habitation sites in resource-rich river valleys and diminishing use of upland habitats [7]. By AD 1300, the remains of maize, squash, and eventually the common bean (*Phaseolus*) become ubiquitous in midden deposits of living sites and point to agriculture as an integral part of most subsistence systems [1,8]. The intensification of inter-group conflict is seen in the construction of wooden palisade and moat defenses around tribal villages and Mississippian towns, as well as direct evidence of traumatic injuries on skeletal remains and embedded unnotched arrow points in human bones and thoracic areas of skeletons [9].

Particular elements of this new era, such as intensified warfare and the pervasive fear of violence, undoubtedly put all of these populations under varying degrees of social stress. In addition, increased conflict due to the nucleation of village settlements and reduction of free (i.e., safe) mobility beyond the settlement likely increased pressure on local populations to successfully exploit the main prey species, white-tailed deer [10]. Deer are the most common mammal remains found at late pre-contact sites [11–16]

**Fig 1:**
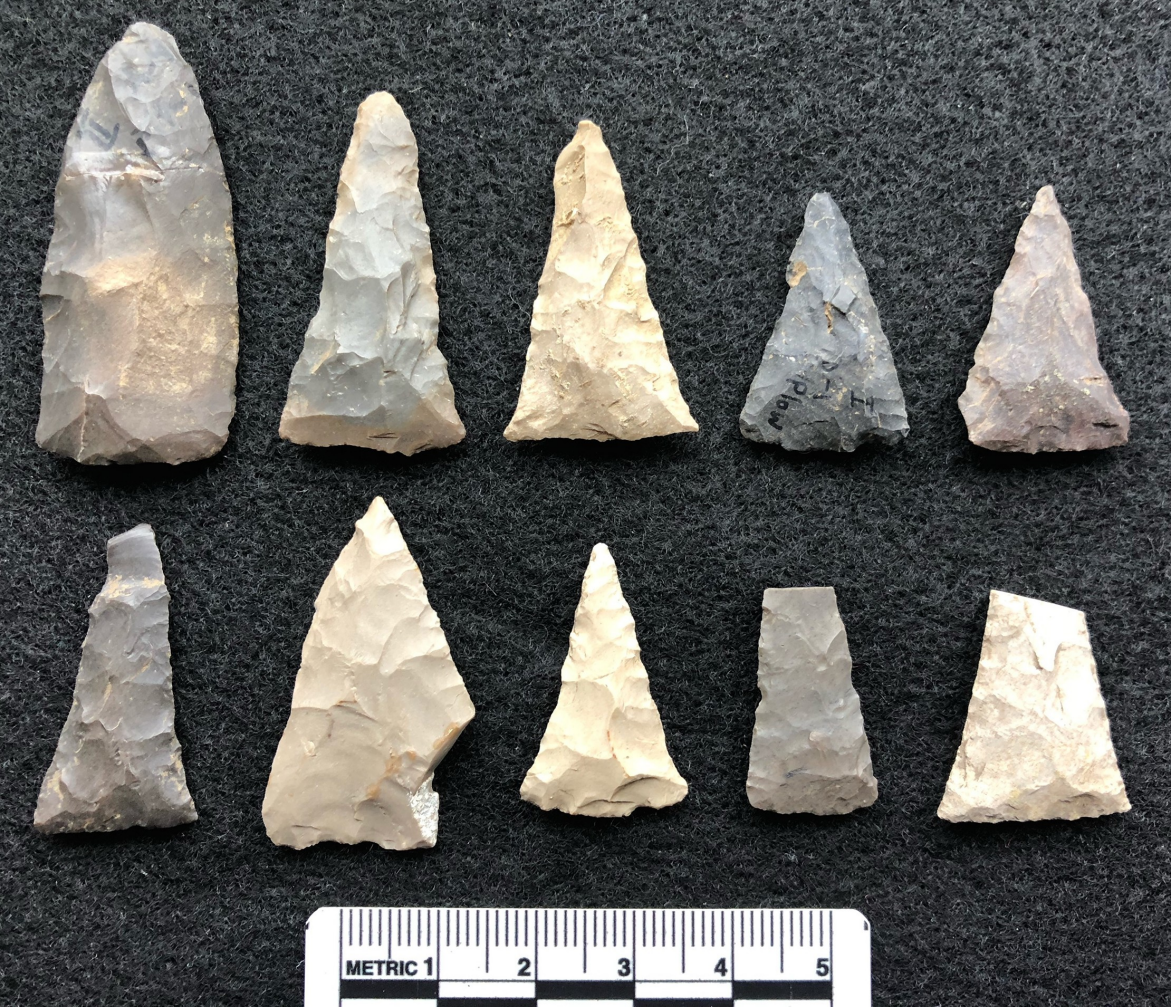
Triangular stone arrow tips (TSAT) (Blain Village, Ross County, Ohio, U.S.A.).

The warfare and hunting that occurred during AD 600–1600 of the North American midcontinent and northeast would have involved the use of small, triangular stone arrow tips (TSAT) [12, 17–19]. The profuse frequency and density of these TSAT is exceptional in the North American archaeological record, and serves as a valuable case for understanding the global phenomenon of “lithic miniaturization” that occurs during the late Pleistocene and throughout the Holocene (**Fig 1**) [20]. Hafted onto arrow shafts, prehistoric archers would have shot TSAT, often referred to as ‘‘Levanna points” or ‘‘Madison points” [21,22], via the bow and arrow [23–25].

Recent analyses of large samples of TSAT from the Eaton site (AD 1550 [12,18]) and Blain Village (AD 1280–1320, **S1 Appendix of S1 Table**) support the hypothesis that TSAT were designed with both warfare and hunting in mind. With respect to warfare, the high length-to-width ratios and low thickness-to-length ratios of TSAT would have facilitated breakage upon impact [26,27], potentially causing more damage to enemy combatants [28,29]. With respect to hunting, by constraining TSAT cross-sectional area [30] to less than 275 mm^2^ –no more than 50 mm in basal width and 11 mm in thickness–prehistoric hunters could have successfully shot between a deer’s ribs to penetrate its thoracic cavity, puncturing the lungs and causing internal bleeding and collapse of the animal [12; 31]. To clarify, 275 mm^2^ = 50 mm x 11 mm; 50 mm is reported by Engelbrecht ([12]:763), who states “the distance between ribs on a deer depend on its age and sex, but for an adult deer, the ribs would be roughly 2.5 cm and 5 cm apart”; 11 mm is also reported by Engelbrecht ([12]:763), citing Guthrie’s [31] experiments, the latter finding points under 10 mm or 11 mm in thickness “more often passed between the ribs and penetrated the thorax” than did thicker points.

**Fig 2:**
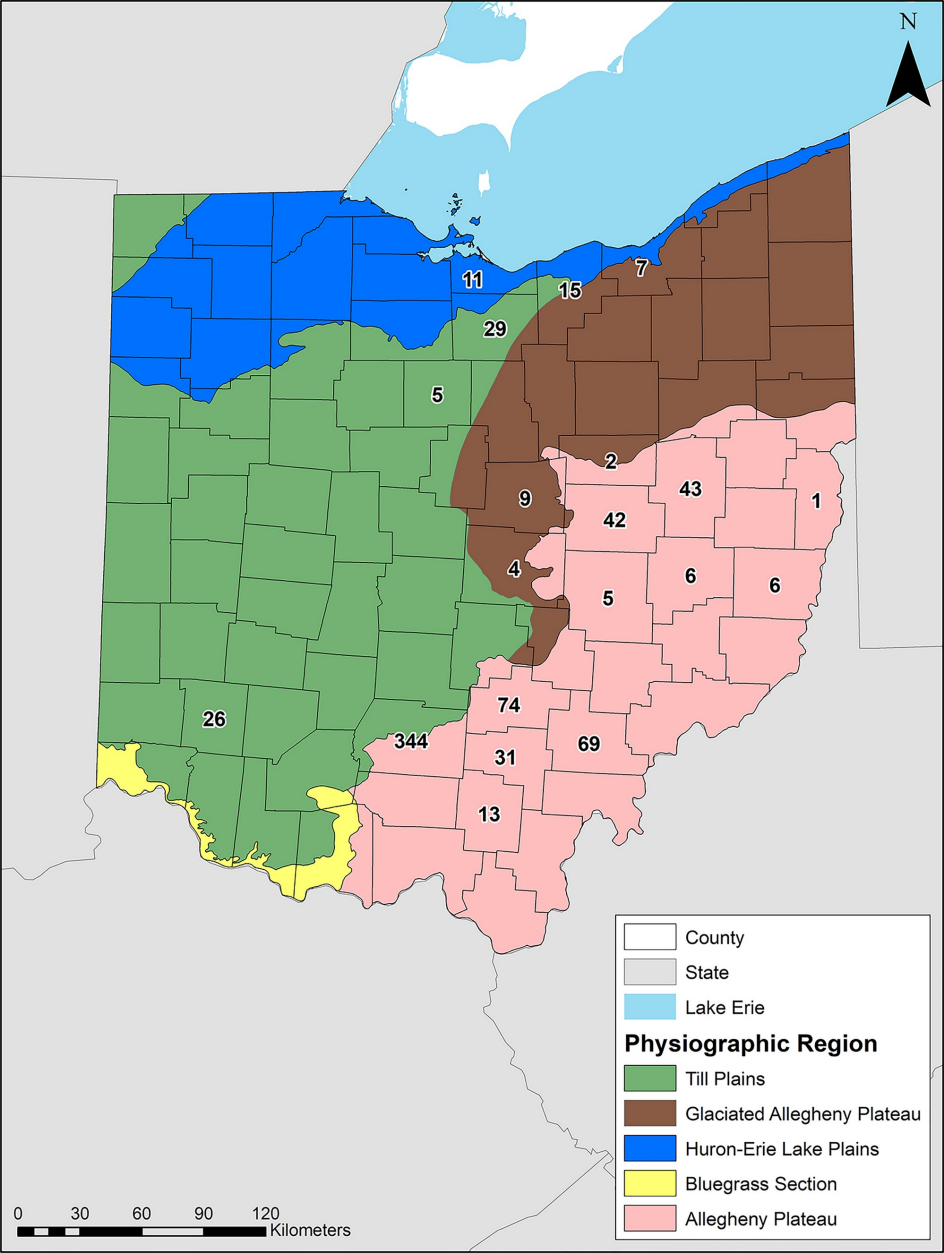
Distribution of the archaeological TSAT sample (n = 742) and physiographic regions across Ohio, U.S.A. This map was created in ArcGIS.

Beyond the cross-sectional threshold of 275 mm^2^, however, little is known as to what extent people selected for TSAT size during the late pre-contact period. Did social stress during this period potentially influence the size of their chief weaponry? The examination of modern metal or carbon projectiles, arrows, and arrowheads by bow hunters and academic researchers has demonstrated that smaller cross-sectional areas penetrate deeper into a target than do larger ones [32–36] (**S1 Appendix**). Do TSAT behave in a similar manner? And if so, did archers under social stress select for smaller, more deeply penetrating stone arrowheads? To answer these questions, we conducted archaeological experiments using replica TSAT, followed by analyses of a large sample (n = 742) of late pre-contact TSAT across several different environmental zones **(Fig 2).** If archers during the late pre-contact period selected for smaller, more deeply penetrating arrow tips, then we can make two predictions. First, replica TSAT should behave similarly to modern arrow tips, showing an inverse relationship between size and penetration under the cross-sectional threshold of 275 mm^2^. Second, the archaeological sample should be significantly skewed towards the smaller values of the 0 to 275 mm^2^ cross-sectional range with a central tendency significantly smaller than 68.75 mm^2^, which is the first-quartile value between 0 and 275 mm^2^.

## Materials and methods

### Production of experimental TSAT

**Fig 3:**
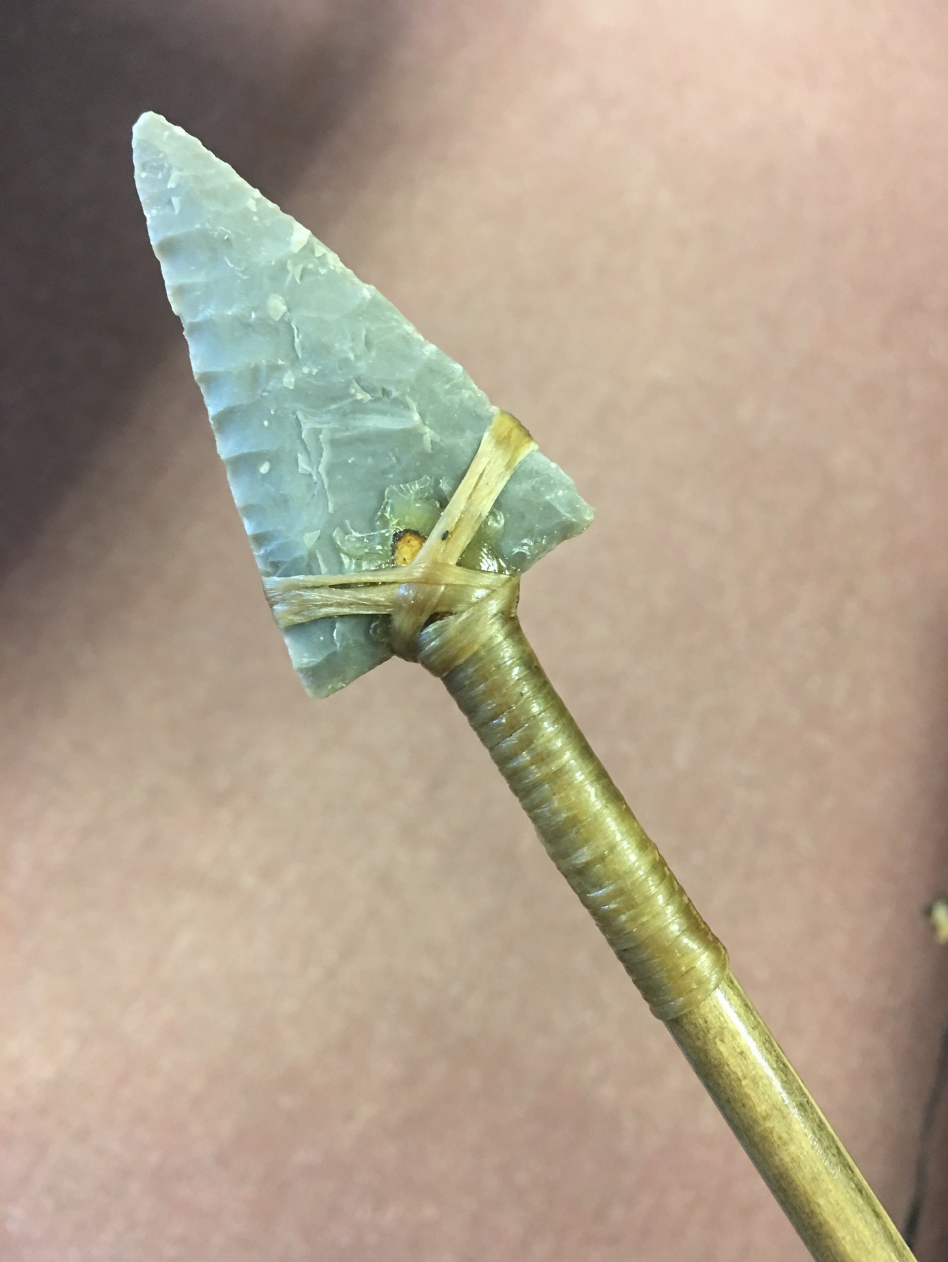
An example of an experimental TSAT (specimen #33).

M.I.E. knapped thirty-five flaked stone arrowheads using Texas Georgetown Chert (**S1 Data**) (**Fig 3**). This Edwards Formation chert is an excellent, flaw-free stone comparable to many high quality cherts across North America, including Ohio. There is no evidence to our knowledge that North American pre-contact knappers used any other toolstone (e.g. obsidian, quartzite, etc.) for their stone points beyond chert. First, M.I.E. knapped flakes from chert nodules via hard-hammer direct percussion. Second, he produced preforms using soft-hammer direct percussion. Finally, he finished the triangular arrowheads using pressure flaking. The experimental arrowheads ranged in cross section (width * thickness/2) from 25 to 138 mm^2^ with a mean of 67 mm^2^ and a median of 69 mm^2^ and were normally distributed (skewness = 0.90; Shapiro-Wilk test W = 0.94, p = 0.07) (**S1 Data**). The masses of the points ranged from 1 g to 18 g with a mean of 6 g and a median of 5 g.

### TSAT hafting

M.W. hafted all arrowheads in an identical fashion to 105, 5/16” diameter Port Orford cedar shafting (prefletched) (**Fig 3**). He also used additional materials such as synthetic polyurethane sinew, “Ferr-L-Tite” hot-melt adhesive, and nitrocellulose lacquer for a finish coat to increase haft durability. The hafting process, which involved six steps, required the utilization of several hand tools including files/rasps, a small knife, sand papers of varying grit, and a small “Sterno” heat pot. The first step required tapering the shafts to accommodate the thickness, width, and length of the projectile to be hafted. The second step involved cutting the socket in which the base of the projectile was to be seated. The third step involved trimming and heat-treating the socket area, followed by dry-fitting the projectile and balancing it via hand-spinning. The fourth step involved fixing the projectile in the socket. This step required simultaneously heating the shaft, the projectile, and the hot-melt adhesive. The process of fitting and balancing was repeated, and, with the application of the adhesive, M.W. firmly fixed the projectile and trimmed and smoothed any excess adhesive. After letting the adhesive set, cool, and cure, M.W. completed the fifth step: applying the sinew wraps. The sinew wrap consisted of a simple “loop-and-tail” whip knot. After wrapping, M.W. trimmed the tails, and mildly reheated the entire projectile for the purpose of minor readjustments and smoothing. Lastly, the sixth step involved the application of a nitrocellulose lacquer coat, simulating hide glue, to improve overall projectile durability.

The finished hafted projectiles ranged from 27 g to 44 g with a mean of 32 g and a median of 31 g (**S1 Data**).

### Experimental procedure

Our experiment here closely replicates the practices described in other ballistics studies conducted at the Kent State University Experimental Archaeology Laboratory [37–42]. This laboratory is a controlled experimental indoor setting used in order to systematically evaluate projectile performance. We used a compound bow mounted on a bow-tuning machine, the Spot-Hogg “Hooter Shooter”. The compound bow used in this experiment was the Microburner MX model produced by PSE (Precision Shooting Equipment), Inc., with a draw weight of 29 lbs.

We fired at a stationary target. The distance between the target and bow was approximately 2.75 meters, thus allowing sufficient room for the specimens to travel once fired without losing speed or dropping. To protect participants and observers from any airborne debris during the experiment, a safety wall separated the target from the rest of the laboratory. We fired the hafted projectile specimens into blocks of moist clay containing crystalline silica, which has been used as an ethical substitute for meat and tissue in other studies [37, 39, 43–45]. Key et al. ([39]:2–42) write,

“High-speed video analyses and depth of penetration tests suggest that, dynamically, clay can be used as a suitable substitute for meat during experimental archaeology tests with stone points, but not for modern composite arrows. That is, for studies concerned with the performance of reasonably large projectile tips (such as those often observed in the Palaeolithic archaeological record), clay may be used as reliable proxy for meat.”

Given that our experiment used stone points hafted onto wooden shafts, rather than field tips hafted onto composite shafts, clay in this case is a satisfactory target for relative comparisons and for *potential* penetration depths on animals or human targets. It is also noteworthy that Karger et al.’s [46] experimental results caused them to question the use of gelatin for arrow wounds. Thus, much more testing is required to establish the similarity and differences of meat, clay, and gelatin; this topic should be a focus for experimental archaeologists in future years.

The clay was terracotta low-fire earthenware clay, commonly referred to as “potter's clay” [42]. The clay comes packaged in rectangular 11.34 kg (25 lb) blocks covered in thin, clear plastic with measurements of 14 cm wide x 15.5 cm deep x 28 cm long. We placed the clay blocks on the wooden target three wide, two deep, and standing vertically, with one additional block lying horizontally on top of the middle block [42].

To measure velocity, we used a Gamma Master Model Shooting Chonograph throughout the experiment. The device is able to measure velocities from approximately 9.14 meters per second (mps) to up to 2133.6 meters per second (mps). The Chrony readings on occasion result in “error” if there is a change in sunlight, cloud cover, or some other minor variable. As a result, we recorded 31 of 35 possible stone point velocity readings (**S1 Data**). The recorded impact energies ranged from 11.2 J to 29.1 J, with a mean of 16.3 J and median of 15 J (**S1 Data**). The range is due to Chrony reading measurement error; the inter-quartile range (14.0 J to 16.1 J) illustrates that the impact energies are approximately the same regardless of point mass (**S1 Data**), which is expected given all projectiles were pulled to the same draw length.

With respect to target penetrability, each arrowhead was shot into a clay target once. We recorded penetration depth into the clay target for each shot. We measured this variable by marking the shaft with a pen at the location at which the shaft was first exposed in the clay target [42]. Once we removed a specimen from the target, a tape measure was used to measure from the pen mark on the shaft to the tip of the point (**S1 Data**).

### Archaeological sample

Once the experiments were complete and the relationship between point size and penetration depth was modeled, we analyzed a large sample (n = 742) of archaeological TSAT. These specimens represent every TSAT previously curated in the Department of Anthropology at Kent State University that possessed the necessary measurements–basal width and thickness–to calculate cross-sectional area. As of August 2019 these specimens, with the exception of Blain and Kramer, are curated by the state repository, the Ohio History Connection. The points come from a variety of sites across Ohio (**S1 Data**), which themselves are located in different environments across the state (**Fig 2**). These locations formed three clusters: a southern cluster, represented by sites from the Allegheny Plateau and Till Plains; a mid-state cluster, represented by sites from the Allegheny Plateau and glaciated Allegheny Plateau; and a northern cluster, represented by sites from the glaciated Allegheny Plateau, the Till Plains, and the Huron-Lake Erie Plains. The point sample also occurs across archaeological cultural boundaries and ceramic traditions, such as Fort Ancient, Clover, Eastwall/McFate, Whittlesey, and Sandusky [47–50].

No permits were required for the described study, which complied with all relevant regulations. All specimens are curated at the Ohio History Connection or Kent State University.

## Results

### Experimental modeling

**Fig 4:**
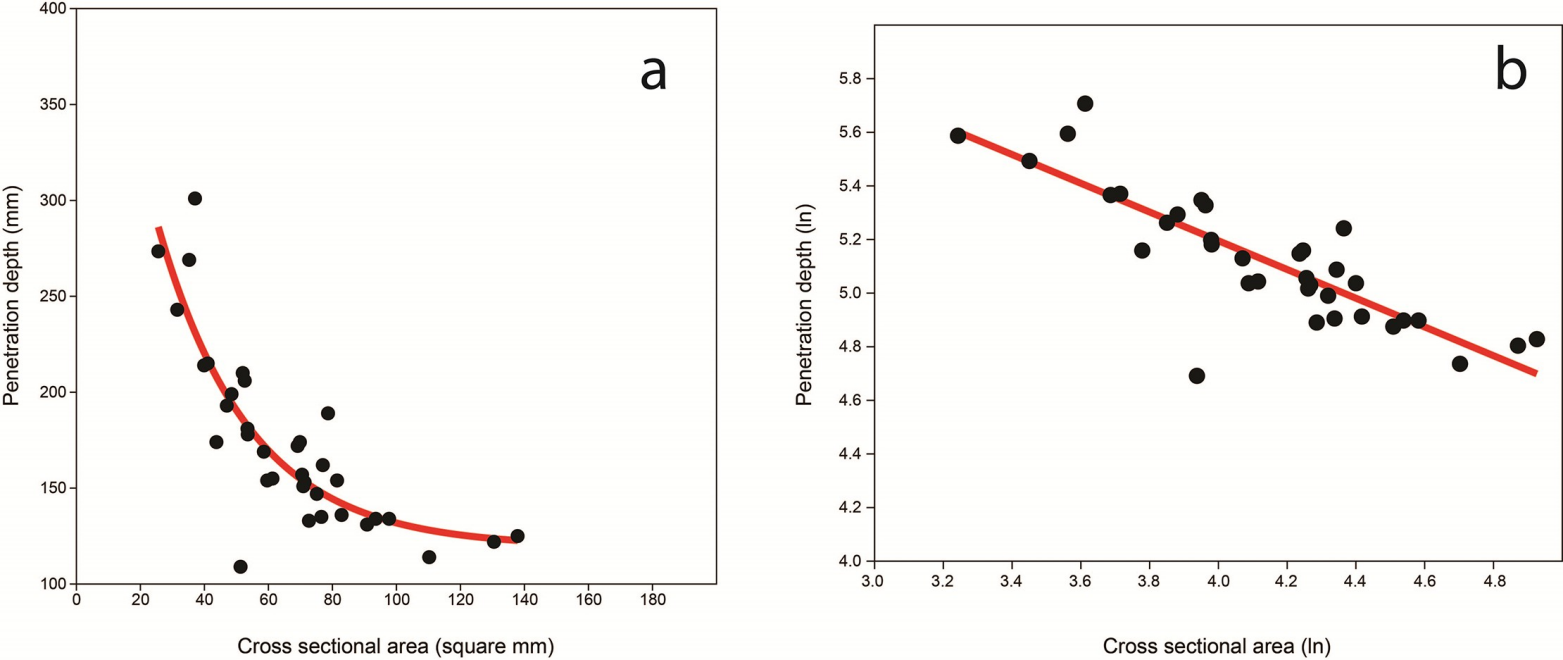
Models of point cross-sectional area and penetration depth: Exponential decay model (a); Ordinary least squares fit of the logarithmically-transformed data (b).

We modeled the relationship between TSAT cross-sectional area and penetration depth using a series of nonlinear and linear best-fit lines. We used minimal Akaike's Information Criterion (AIC) score to determine the best fit. An exponential decay model had the lowest AIC (18858) and had a negative decay rate of -0.034. The exponential decay model indicates that TSAT with cross-sections between about 70 and 25 mm^2^ demonstrate the greatest increases in penetration depth, with smaller cross sections exhibiting exponential increases in penetration depth (**Fig 4A**). The TSAT cross section and penetration depth relationship can also be modeled by log-transforming both variables and using an ordinary least squares linear fit **(Fig 4B)**. The linear fit is significant and explains approximately 69% of the variation in the data (r^2^ = 0.69; slope = -0.54, y-intercept = 7.34). This model indicates a sublinear allometric relationship with penetration depth decreasing at a rate of approximately 1/2 with that of cross section area. In other words, TSAT penetration depths decrease faster as cross section area increases.

### Archaeological analyses

**Fig 5:**
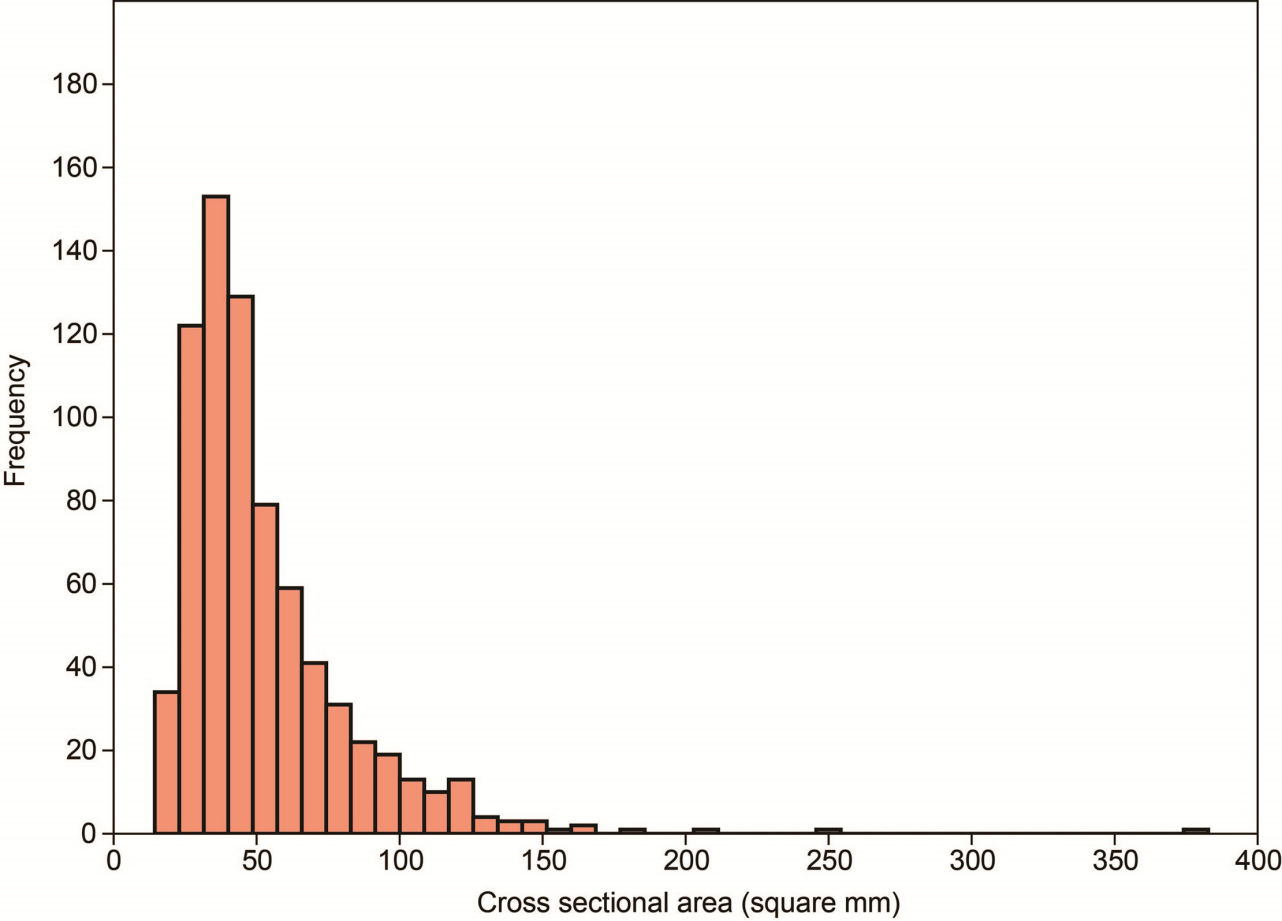
Histogram of point cross-sectional area.

**Fig 6:**
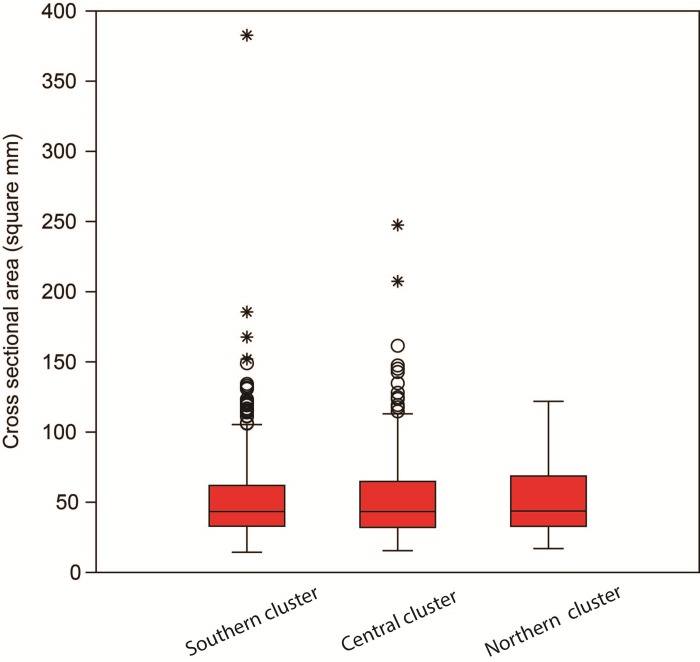
Box plot of point cross-sectional areas by geographic cluster.

All archaeological specimens in our sample were under 275 mm^2^ in cross-sectional area, except for one. They ranged from 14 to 383 mm^2^ with a mean of 52.5 mm^2^. The distribution of cross section area measurements of the archaeological samples does not conform to an underlying normal distribution (Shapiro-Wilk test W = 0.77, p<0.000) as it displays significant kurtosis (21.5) the peak occurring at a median of 43.4 mm^2^ (**Fig 5**). This sample median was significantly smaller than 68.75 mm^2^, which is the first-quartile value between 0 and 275 mm^2^ (Wilcoxon test: *W* < 0.000, z = 14.997, p<0.000). If the sample is divided into three clusters of points, each located in a different environmental zone, the clusters cross sectional areas do not differ from each other (Kruskal-Wallis test H = 0.39, p = 0.82) (**Fig 6**).

## Discussion

When taken together, these experimental and archaeological results are consistent with the hypothesis that prehistoric archers during the North American late pre-contact period selected TSAT sizes that maximized penetration, and that this selection occurred across environmental and cultural boundaries. Similar to modern metal arrow tips, our experiments using replica TSAT confirmed the inverse relationship between size and penetration. Our archaeological analyses subsequently demonstrated that rather than produce a variety of TSAT sizes that simply complied with the 275 mm^2^ threshold necessary to effectively dispatch deer, prehistoric people appear to have significantly winnowed their TSAT to an adaptive peak far beyond this threshold [51,52]. When the size analyses presented here are considered alongside previously published TSAT shape analyses, the latter suggesting that TSAT were designed to break on impact to increase wound trauma [12,17], and analyses of TSAT archaeological context [18], a strong case can be made that people during the late pre-contact period were concerned with ensuring the kill of both prey and enemy combatants alike. The selection of such extreme reliability and performance in weaponry can be cogently linked to the social stress of the period, caused by increased population size, territoriality, and warfare.

The case for an adaptive evolutionary change must always remain an inference. Here, because our experimental data show a clear functional advantage for smaller points, our archaeological data demonstrates a tendency for smaller points, and the stressful context these archaeological data are from can be directly linked to a plausible human adaptive response [53–55], we infer that smaller point sizes were selected for improved penetration. However, as we have suggested elsewhere ([17]:79), while the morphology and functional properties of TSAT appear to be consistent with an intended design involving hunting and warfare, it is important to acknowledge that point size may be instead only incidental, the result of these items interacting with a complex, multi-component weapon system. For example, their size may be conducive to hafting to a narrow, aerodynamic arrow shaft ([17]:79). Or, we fully acknowledge that the emergence of TSAT during the North American pre-contact area could potentially be the product stochastic processes such as stylistic drift or copying error [e.g. 56–61], production economizing behavior, or perhaps even general social preference for different point forms. Future research should focus on acquiring additional lines of evidence to support the adaptive TSAT hypothesis ([17]:80). Following Bebber et al ([17]:79), none of this is to say that TSAT would not have provided benefits to pre-contact hunting and warfare, “only that the ultimate source of these benefits is difficult to pin down.”

The emergence of small stone tools during North America’s first millennium AD, however, is not unique in the archaeological record. Indeed, lithic “miniaturization” is one the archaeological record’s most pervasive and variable technological features [20]. As such, our study has potential to shed light on a global technological phenomenon that occurred repeatedly during the Late Pleistocene and Holocene. Several factors likely influenced toolmakers’ decisions to miniaturize toolkits including efforts to exploit raw materials more efficiently, to produce interchangeable parts and composite tools, to reduce a wider range of rock types, and to increase the aerodynamic and ballistic properties of smaller and lighter armatures. However, on a relatively broader level, lithic miniaturization and the functional benefits it provides has been usually linked to three hypotheses: the emergence of modern cognition; increased mobility; and demographic shifts related to increased territoriality, population size increase, and inter-group conflict [20, 62–64]. Of these three hypotheses, in recent years the demographic hypothesis has seen a substantial increase in support.

Tryon and Faith [65], for example, hypothesize that increasing site occupation intensity at Nasera rockshelter in Tanzania ~40 ka, through processes connected with wider population pressure, resulted in decreased access to raw materials and increased reliance on local rocks. These demographic processes placed greater pressure on humans to conserve raw material by using increasingly miniaturized lithic reduction strategies. Similarly, Eren et al. [66] argue that increased lithic miniaturization structured around bipolar reduction may relate to population pressure and the demands placed on local resources at Mumba rockshelter in Tanzania. Both models link demographic shifts to resource scarcity and greater lithic miniaturization. Marean [67] argues that southern African Late Pleistocene populations’ consistent use of marine resources resulted in reduced mobility, larger group size, population packing, smaller territories, and increased reliance on composite technologies built from miniaturized lithic components. Drawing on data from the Indian subcontinent, Petraglia et al. [68] maintain that decreased ecological productivity ~ 35–28 ka led to population packing in ecological refugia and shifts towards miniaturized technological systems designed to hunt key resources in shrinking favorable ecological zones [cf. 69]. Finally, Mackay et al. [70] similarly hypothesize that population coalescence events between ~130–12 ka in southernmost Africa drove periods of increased lithic miniaturization (which they link indirectly to changes in subsistence procurement strategies).

We find similar support in our New World case study here that demographic processes of fragmentation of interacting metapopulations likely led to lithic miniaturization. This is important because it can be difficult to completely discount other factors such as raw material scarcity or increased mobility that may have contributed to the production of small stone tools in earlier periods in the Old World. Significantly, however, we can rule out both of these factors in the late pre-contact of the North American midcontinent and northeast, leaving only demography as the predominant causal factor. Raw material scarcity can be discounted as a factor because North American late pre-contact groups had access to a range of rocks from which to make tools. Increased mobility can likewise be discounted as a factor because during the late pre-contact period mobility decreases as a result of sedentism and isolation of social group [47, 71]. Instead, our case study shows how, like in India and southern Africa, humans responded to increased demographic pressures by downscaling lithic production. In order to adapt to and survive demographic changes in a socially stressful period of increased territoriality and warfare, rather than “going big” North American late pre-contact toolmakers and archers “went small”. They selected for an extreme level of lithic miniaturization that provided a vital functional advantage: ensuring the killing of prey and enemy combatants alike.

## Supporting information

S1 Appendix (DOCX)

S1 Data (XLSX)
