# Supplementary material for: Miniaturization optimized weapon killing power during the social stress of late pre-contact North America (AD 600-1600)
Source: PLoS One. 2020 Mar 17;15(3):e0230348. doi: 10.1371/journal.pone.0230348 (PMC7077820; doi:10.1371/journal.pone.0230348)
Supplement: S1 Appendix — (DOCX) [file pone.0230348.s001.docx]

Supplementary Information for

Miniaturization optimized weapon killing power during the social stress of late pre-contact North America (AD 600-1600)

Anna Mika, Kat Flood, James D. Norris, Michael Wilson, Alastair J.M. Key, Briggs Buchanan, Brian Redmond, Justin Pargeter, Michelle R. Bebber, Metin I. Eren

Email: [meren@kent.edu](mailto:xxxxx@xxxx.xxx)

**This PDF file includes:**

Supplementary text

Table S1

Legends for Datasets S1 to S2

SI References

**Other supplementary materials for this manuscript include the following:**

Dataset S1

Supplementary Information Text

 *Dating Blain Village* Given the early dates generated by previous radiocarbon dating (980-725 B.P.) (1), and the site’s role in debates on Triangular Stone Arrow Points (TSAT) (2), we felt it necessary to re-date the Blain Village site, for three reasons: first, to better establish Blain Village’s place in the archaeological record of the Scioto River Valley; second, to better understand its relationship to other Late Woodland and Late Pre-contact sites in the region; and three, to better frame how the tool types used by the inhabitants of Blain Village, such as the triangular point discussed here, fit into population driven patterns seen during the Late Pre-contact era. While these new dates are part of a broader, long-term re-investigation of Blain Village, we present them here for the first time.

Bone specimens were randomly selected from a sealed feature (Feature 2). Feature 2 was previously dated to 1035 ± 155 B.P. Faunal remains excavated from this feature have been stored in archival boxes since the excavation. Six specimens were pulled for sampling, 2 deer rib bones, 2 deer long bones, 1 deer “irregular” bone, and 1 bird long bone (**Table S1**). In order to attain the most accurate consensus possible, the bone samples were randomly divided into groups, and then sent to three different radiocarbon dating facilities: The University of Georgia Center for Applied Isotope Studies (CAIS), International Chemical Analysis, and Beta Analytic.

The results of these analyses are overall very consistent (except for specimen 4 which likely was the result of admixture when the pit was initially dug for use as a trashpit). These results place the Blain Village occupation at the temporal threshold between the end of the 13^th^ and the beginning of the 14^th^ century AD. These dates are much later in time than the dates originally asserted by Prufer and Shane (1).

*Projectile penetration mechanics*

The depth of penetration achieved by a stone projectile when thrown, cast, or fired at a target material is dependent on the form and mass of the projectile point and shaft, its flight path and velocity, any variation in air resistance, and the properties of the material pierced (3-8). For the experiments described below, flight path, bow draw-length, air resistance, and pierced material are held constant, and the independent variable under consideration is projectile point size, specifically cross-sectional area (9).

The principal means through which cracks (i.e. cuts, fractures, wound cavities) propagate during stone projectile use is through ‘pedalling’, a separation process whereby parts of the target material is displaced by the point and cracks initiate to permit the projectile to pass through (3). Indeed, after an initial period of deformation (whereby a ductile material bends in response to loads applied by the projectile point), a target material will move to either side of the projectile so long as there is sufficient force for perforation and continued movement of the point through the material. Energy (work of penetration) is required to both incrementally create the crack as the projectile progress through the target material and provide sideways compression on the material (3). As with many cutting processes (10), ease of cut and amount of material deformed is directly related to the amount of cutting stress (force / unit area) created between a sharp edge and worked material. The greater the cutting stress, the more likely it is that the material bonds will fail, a crack will form and the cutting edge will be able to move into and separate a material.

Assuming the form of the projectile’s shaft is held constant, the mass of a projectile’s point has potential to significantly impact on material deformation and damage (and in turn penetration) due to its direct relationships with a projectile’s energy (3, 6). Certainly, the mechanical efficiency of a projectile is dependent on its kinetic energy, and subsequently work of penetration, which, when velocity is held constant, is dependent on its mass (5). That is, when projectiles are fired with the same velocity, the greater the mass of the projectile head, the greater its kinetic energy at the point of impact. This energy then serves to produce a crack in the material, and the greater the energy the greater the amount of material able to be split in a single ‘shot’ (6, 11). A linear relationship between greater point mass, kinetic energy, and in turn, projectile penetration, cannot however be automatically assumed due to the allometric scaling observed between point size, surface area, cross section, and point mass. The relationship between a projectile’s mass and principal cross sectional area is referred to as its ‘sectional pressure’ (mass divided by cross sectional area), and is a straightforward means of determining a projectile’s effectiveness (3). Essentially, the greater the sectional pressure value then the deeper the penetration of the point is likely to be. Cross sectional area significantly impacts on penetration depth as it determines how much target material is required to be split and pushed to the side as the point produces a wound cavity. The more materials required to be split and deformed, the greater the energy required for a set depth of penetration to be achieved.

All points used in the experiments described here were near identical in pyramidal 3D shape. While the relative ‘pointedness’ of a pyramid point (length relative to width) has potential to impact on requires forces prior to perforation, with more elongated pointed forms requiring lower loads (3,4), shape is assumed here to be consistent in this experiment. Hence, form related differences between points are primarily an issue of scaling and incremental increases/decreases in point size. For a pyramid point, each of the two lateral edges of the point creates its own pedalling cut in the material (3,4). This means that larger points have greater lengths of sharp edge making contact with a target material, which due to their splayed shape away from the points tip, results in larger amounts of target material being cut (i.e. a larger wound cavity). This inevitably increases material resistance, increases the rate of point deceleration, reduced the cutting stress achieved per unit area of sharp edge, and reduces depth of penetration for a given amount of kinetic energy. Particularly thin points, such as those used here, require low forces for penetration due to the relative small surface areas forced through a target material (4). Thus, increases in point size can have a relatively large impact on the amount of point surface area required to be forcibly pushed through the target material. Moreover, and as demonstrated by Swain et al. (7), penetration depth has previously been demonstrated to directly correlate with projectile diameter. In other words, the smaller the point the greater the depth of penetration is likely to be, simple as a function of the amount of material required to be deformed. Increases in point size additionally have potential to negatively impact on penetration depth as larger point surface areas act to increase the air resistance (drag) experienced by a projectile prior to impact, in turn reducing the kinetic energy present at the point of impact (5).

**Table S1. Radiocarbon samples and dates from Blain Village, Ohio.**

| Sp # | Species | Part | Feature | mass (g) | Lab | Dates (BP) | Dates (AD) |
| --- | --- | --- | --- | --- | --- | --- | --- |
| 1 | Bird | Long Bone | F-2 | 1.9 | CAIS | 650+/-20 | 1280-1320 cal AD |
| 2 | Deer | Rib | F-2 | 3 | CAIS | 630+/-20 | 1300-1340 cal AD |
| 3 | Deer | Long bone | F-2 | 4.5 | CAIS | 630+/-20 | 1300-1340 cal AD |
| 4 | Deer | Rib | F-2 | 4.7 | ICA | 3530 +/- 30 | 1950-1760 BC |
| 5 | Deer | Irregular bone | F-2 | 5.3 | ICA | 630 +/- 30 | 1280-1400 cal AD |
| 6 | Deer | Long Bone | F-2 | 6.4 | Beta Analytic | 670 +/-30 BP | 1274-1320 cal AD |

Dataset S1 (separate file). Experimental data.

**Dataset S2 (separate file).** Archaeological data.

**References**

1. O. Prufer, O. Shane. Blain Village and the Fort Ancient Tradition in Ohio (Kent State University Press, 1970).

2. M. Bebber, S. Lycett, M. Eren. Developing a stable point: evaluating the temporal and geographic consistency of Late Prehistoric unnotched triangular point functional design in Midwestern North America. Journal of Anthropological Archaeology 47, 72-82 (2017).

3. T. Atkins. The Science and Engineering of Cutting. (Butterworth-Heinemann, 2009).

4. E. Cheshire, M. Rossi, T. Atkins. Perforation of sheets by pyramidal weapons such as arrowheads. International Journal of Impact Engineering 35, 457-470 (2008).

5. B. Cotterell, J. Kamminga. Mechanics of Pre-Industrial Technology (Cambridge University Press, 1990).

6. N. Maiden. Ballistics reviews: mechanisms of bullet wound trauma. Forensic Science, Medicine, and Pathology 5, 204-209 (2009).

7. M. Swain, D. Kieser, S. Shah, J. Kieser. Projectile penetration into ballistic gelatin. Journal of the Mechanical Behavior of Biomedical Materials 29, 385-392 (2014).

8. Y. Wang, X. Shi, A. Chen, C. Xu. The experimental and numerical investigation of pistol bullet penetrating soft tissue simulant. Forensic Science International 249, 271 – 279 (2015).

9. M. Sisk, J. Shea. Experimental use and quantitative performance analysis of triangular flakes (Levallois points) used as arrowheads. Journal of Archaeological Science 36, 2039-2047 (2009).

10. A. Key. Integrating mechanical and ergonomic research within functional and morphological analyses of lithic cutting technology: key principles and future experimental directions. Ethnoarchaeology 8, 69-89 (2016).

11. N. Rozen, I. Dudkiewicz. “Would ballistics and tissue damage” in Armer Conflict Injuries to the Extermities, A. Lerner, M. Soudry, Eds. (Springer-Verlag, 2011), pp. 21-33.
